# The deubiquitinase USP11 promotes ovarian cancer chemoresistance by stabilizing BIP

**DOI:** 10.1038/s41392-021-00580-w

**Published:** 2021-07-14

**Authors:** Xiaolin Zhu, Yiping Zhang, Qingyu Luo, Xiaowei Wu, Furong Huang, Tong Shu, Yong Wan, Hongyan Chen, Zhihua Liu

**Affiliations:** 1 State Key Laboratory of Molecular Oncology, National Cancer Center/National Clinical Research Center for Cancer/Cancer Hospital, Chinese Academy of Medical Sciences and Peking Union Medical College, Beijing, 100021 China; 2 Key Laboratory of Cancer and Microbiome, National Cancer Center/National Clinical Research Center for Cancer/Cancer Hospital, Chinese Academy of Medical Sciences and Peking Union Medical College, Beijing, 100021 China; 3 Department of Gynecological Oncology, National Cancer Center/National Clinical Research Center for Cancer/Cancer Hospital, Chinese Academy of Medical Sciences and Peking Union Medical College, Beijing, 100021 China; 4 Department of Obstetrics and Gynecology, Department of Pharmacology, The Robert H. Lurie Comprehensive Cancer Center, Northwestern University Feinberg School of Medicine, Chicago, 60611 IL USA

**Keywords:** Cancer, Cell biology

**Dear Editor**,

Chemoresistance is a major problem in the treatment of ovarian cancer patients and leads to poor prognosis, but its underlying mechanism remains elusive. Accumulating evidences have found that the aberrant regulation of the protein ubiquitination pathway plays an essential role in ovarian cancer chemoresistance.^1–3^ However, it is unclear whether and how deubiquitinase-USP11 involves in ovarian cancer chemoresistance.

We previously reported that USP11 suppresses cancer cell apoptosis via its direct substrate XIAP.^4^ These findings motivate us to further investigate the role of USP11 in chemoresistance. We firstly examined the protein level of USP11 in 70 ovarian cancer tissues using immunohistochemistry (IHC) staining. We found that the USP11 expression is significantly higher in the chemoresistant samples than that in chemosensitive samples (Fig. 1a and Supplementary Fig. S1a). Remarkably, the higher expression of USP11 correlated strongly with shorter overall and progression-free survival (Fig. 1b and Supplementary Fig. S1b). These results suggest that USP11 may act as a predictor for chemoresistance and poor prognosis of ovarian cancer patients.

**Fig. 1:**
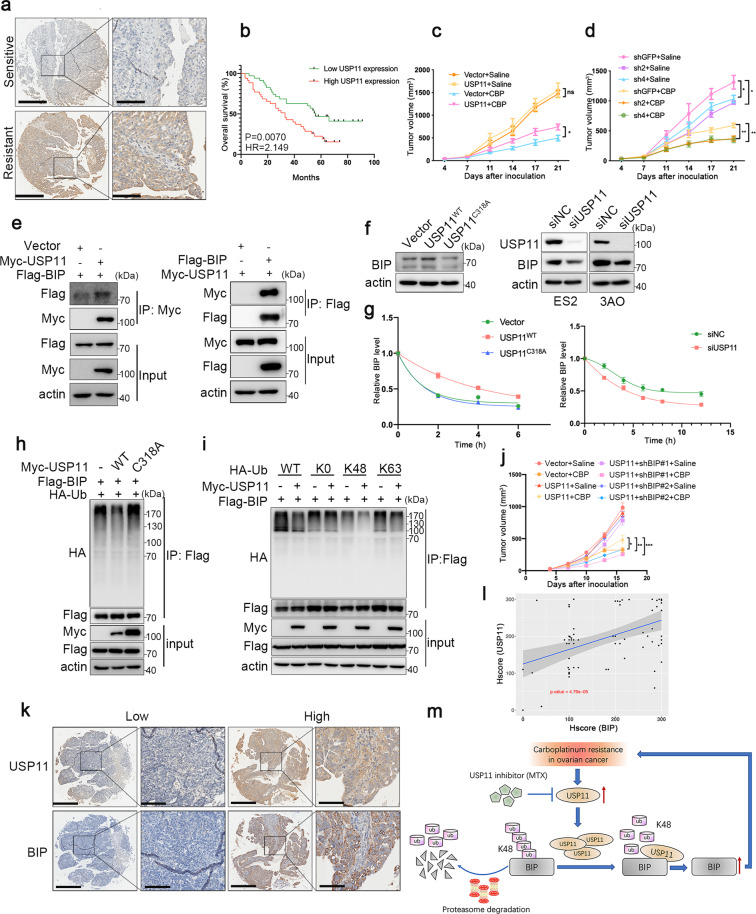
USP11 stabilizes BIP and drives chemoresistance in ovarian cancer. **a** Representative images of IHC staining of USP11 in platinum-resistant or platinum-sensitive ovarian cancer tissues. **b** Kaplan-Meier analysis of ovarian cancer patients’ overall survival grouped by low or high expression of USP11. *P*-values were determined by log-rank test. **c** Tumor volumes of USP11-overexpressed and control ES2 cells without or with CBP treatment. *n* = 6 mice per group. **d** Tumor volumes of USP11-depleted and control 3AO cells without or with CBP treatment. *n* = 6 mice per group. **e** Interaction between ectopically expressed USP11 and BIP was validated by co-immunoprecipitation in HEK293 cells. **f** Left panel: Immunoblotting analysis of BIP expression in ES2 cells transfected with wild type USP11^wt^, mutant USP11^C318A^, and vector control. Right panel: Immunoblotting analysis of BIP expression in USP11-silenced ES2 and 3AO cells and the corresponding control cells. **g** Quantification of protein degradation rates of BIP after USP11^wt^ and USP11^C318A^ overexpression (left panel) and USP11 knockdown (right panel). **h** Immunoblotting to detect the ubiquitination of BIP in HEK293T cells cotransfected with Flag-BIP, HA-Ubiquitin, and Myc-USP11 (WT or C318A). **i** Immunoblotting to detect the deubiquitination of ubiquitin mutants (K0, K48, or K63) linked BIP in HEK293T cells cotransfected with Flag-BIP, Myc-USP11, and HA-Ubiquitin, and its mutants. **j** Tumor volumes of ES2 cells expressing USP11, USP11 together with shRNAs targeting BIP and vector control without or with CBP treatment. *n* = 6 mice per group. **k** Representative images of IHC staining of USP11 and BIP in platinum-resistant or platinum-sensitive ovarian cancer samples. **l** The correlation between USP11 and BIP was analyzed in R. **m** A schematic model showing that USP11 upregulation promotes chemoresistance of ovarian cancer patients. USP11 interacts with BIP and cleaves the K48 ubiquitin chains linked on BIP to prevent the proteasomal degradation of BIP and leads to the elevation of BIP, thereby contributing to the chemoresistance. Data in **c**, **d**, and **j** were analyzed by two-way ANOVA with Bonferroni correction. * *P* < 0.05, **P < 0.01, ***P < 0.001

To determine whether USP11 promotes chemoresistance, we overexpressed USP11 in ES2 and 3AO cells and then treated cells with carboplatinum (CBP). The ectopic expression of USP11 increased the viability, suppressed cell apoptosis, and promoted colony formation capability with CBP treatment (Supplementary Fig. S2a–e). In contrast, USP11 ablation decreased the viability, increased cell apoptosis, and suppressed colony formation capability of ES2 and 3AO cells with and without CBP treatment (Supplementary Fig. S3a–e).

To verify the role of USP11 in vivo, we compared the growth of USP11-overexpressing ES2 and control cells treated with saline or CBP. As expected, CBP treatment markedly attenuated the growth of ES2 cells, while USP11-overexpressed ES2 cells exhibited impaired responsiveness to CBP compared to control cells (Fig. 1c and Supplementary Fig. S4a). While knockdown of USP11 significantly decreased the growth of ES2 cells and promoted the CBP-induced growth inhibition of ES2 cells compared to control cells (Fig. 1d and Supplementary Fig. S4b). Taken together, these data indicate that USP11 is a crucial determinant of resistance to CBP in ovarian cancer cells.

To investigate the molecular mechanism underlying USP11-mediated CBP resistance, we performed immunoprecipitation and mass spectrometry to interrogate the USP11 interactome in ES2 cells. Mass spectrometric analysis showed that USP11 is coimmunoprecipitated with a series of proteins including DDX5, BIP, HSPA1A, PRMT5, and TGM2 (Supplementary Fig. S5a). Among them, we focus on BIP which has reported as being involved in drug resistance in ovarian cancer.^5^ To verify the interaction between USP11 and BIP, we co-expressed Myc-tagged USP11 and Flag-tagged BIP in HEK293T cells and then employed coimmunoprecipitation (Co-IP) assay. As expected, Flag-tagged BIP can be immunoprecipitated by Myc-tagged USP11, and vice versa (Fig.1e). To further consolidate the interaction between USP11 and BIP, we performed a GST pull-down assay with a bacterially expressed GST-tagged BIP. GST-BIP can be pulled down by Flag-tagged USP11 in vitro (Supplementary Fig. S5b). We also validated the interaction between USP11 and BIP using an in vivo Co-IP assay (Supplementary Fig. S5c). Furthermore, we defined the binding region of USP11 on BIP and revealed that the amino acid sequence 503-963 with the C-terminus of USP11 mediating the interaction with BIP (Supplementary Fig. S5d). Collectively, these results suggest that USP11 can interact with BIP directly.

To explore whether USP11 regulates BIP expression level, we firstly transfected exogenous wild-type USP11 (USP11^wt^) and the enzymatically inactive USP11^C318A^ mutant^4^ into ES2 cells and examined the protein level of BIP. USP11^wt^ overexpression effectively increased the protein level of BIP, but not USP11^C318A^. In contrast, knockdown of USP11 decreased the protein level of BIP in ES2 and 3AO cells (Fig. 1f). The mRNA level of BIP was not affected after overexpression or knockdown of USP11(Supplementary Fig. S5e). Mitoxantrone (MTX), a USP11 inhibitor, was shown to attenuate the protein level of BIP (Supplementary Fig. S5f), further illustrating that USP11 regulates BIP protein expression.

To elucidate the mechanism responsible for USP11-dependent regulation of BIP, we further investigate whether USP11 stabilizes BIP as a bona fide deubiquitinase. The effect of USP11 on the half-life of endogenous BIP protein was measured. The results showed that USP11^wt^ overexpression can obviously slow down the rate of degradation compared with vector control, but not USP11^C318A^ (Supplementary Fig. S6a and Fig. 1g left panel). Conversely, USP11 knockdown promotes the degradation of BIP (Supplementary Fig. S6b and Fig. 1g right panel). To identify the pathway leading to BIP stability, we treated ES2 and 3AO cells transfected with USP11 and control siRNAs with 20 µM MG132 and 40 µM Chloroquine (CQ). The decrease of BIP expression was reversed by MG132 treatment, but not CQ treatment, suggesting that USP11 stabilizes BIP via proteasome but not lysosome-mediated pathway (Supplementary Fig. S6c, d). We next examined the effect of USP11 on BIP ubiquitination through a ubiquitination assay. USP11^wt^ overexpression led to a decrease in ubiquitinated BIP, but not USP11^C318A^, while USP11 depletion increased the endogenous BIP ubiquitination level (Fig. 1h and Supplementary Fig. S6e, f), indicating USP11 is direct deubiquitinate for BIP. To further expatiate the type of modification of USP11 deubiquitinating BIP, we examined the ability of wide-type ubiquitin, K0, K48R, and K63R mutant ubiquitin to mediate BIP deubiquitination. An in vivo ubiquitination assay reveals that USP11 reduces K-48 but not K-0 or K-63-linked ubiquitination of BIP (Fig. 1i), suggesting that the K48-linked ubiquitination targeting protein substrates for proteasomal degradation is a regulatory mode for orchestrating BIP expression.

To investigate whether chemoresistance caused by USP11 is dependent on BIP, we knocked down BIP in USP11-overexpressed ES2 cells and then treated them with CBP (Supplementary Fig. S7a). The ectopic expression of USP11 inhibited CBP-induced cell apoptosis, while ablation of BIP can effectively relieve the inhibition of cell apoptosis caused by USP11 (Supplementary Fig. S7b). Xenograft assay showed that CBP treatment dramatically inhibited tumor growth of ES2 cells, while overexpression of USP11 significantly suppressed CBP-induced tumor growth inhibition of ES2 cells. Knockdown of BIP is able to rescue the inhibition of tumor growth with CBP treatment (Fig. 1j and Supplementary Fig. S7c). Altogether, these data suggest that the stabilization of BIP by USP11 inhibits cell apoptosis, promotes chemoresistance in ovarian cancer cells.

To further validate the regulation of BIP by USP11 in clinical specimens, we stained the same 70 human ovarian cancer specimens for BIP expression by immunohistochemistry. Expectedly, tumors with high USP11 expression levels exhibited robust staining for BIP (Fig. 1k). A correlation analysis illustrated that the USP11 expression level was positively correlated with BIP (*P* value = 4.79e−05) (Fig. 1l). Notably, the combined analysis of BIP and USP11 also significantly discriminated the prognosis in ovarian cancer patients (Supplementary Fig. S8a, b).

Collectively, in this study, we demonstrate that USP11 is an important determinant of ovarian cancer chemoresistance. Mechanistically, we identified BIP as a direct substrate for USP11 and linked overexpression of the USP11-BIP axis to chemoresistance in ovarian cancer (Fig. 1m). Thus, targeting the USP11-BIP axis may be a novel strategy to increase the chemosensitivity of ovarian cancer patients.

## Supplementary information

Supplementary Material

## Data Availability

The data sets used and/or analyzed during this study are available from the corresponding author on reasonable request.

## References

[1] Matsuura K (2017). Downregulation of the proapoptotic protein MOAP-1 by the UBR5 ubiquitin ligase and its role in ovarian cancer resistance to cisplatin. Oncogene.

[2] Wu X (2019). MGMT-activated DUB3 stabilizes MCL1 and drives chemoresistance in ovarian cancer. Proc. Natl Acad. Sci. USA.

[3] Wu X (2020). JOSD1 inhibits mitochondrial apoptotic signalling to drive acquired chemoresistance in gynaecological cancer by stabilizing MCL1. Cell Death Differ..

[4] Zhou Z (2017). Regulation of XIAP turnover reveals a role for USP11 in promotion of tumorigenesis. EBioMedicine.

[5] Li W (2014). Cisplatin-induced senescence in ovarian cancer cells is mediated by GRP78. Oncol. Rep..

