# Supplementary material for: The deubiquitinase USP11 promotes ovarian cancer chemoresistance by stabilizing BIP
Source: Signal Transduct Target Ther. 2021 Jul 14;6:264. doi: 10.1038/s41392-021-00580-w (PMC8277857; doi:10.1038/s41392-021-00580-w)
Supplement: Supplementary file 1 — Supplementary Material [file 41392_2021_580_MOESM1_ESM.docx]

Supplementary Materials for

The deubiquitinase USP11 promotes ovarian cancer chemoresistance by stabilizing BIP

Xiaolin Zhu^1^, Yiping Zhang^1^, Qingyu Luo^1^, Xiaowei Wu^1^, Furong Huang^1^, Tong Shu^2^, Yong Wan^3^, Hongyan Chen^1^*, and Zhihua Liu.^1^*

^1^State Key Laboratory of Molecular Oncology, National Cancer Center/National Clinical Research Center for Cancer/Cancer Hospital, Chinese Academy of Medical Sciences and Peking Union Medical College, Beijing, 100021, China;

^2^Department of Gynecological Oncology, National Cancer Center/National Clinical Research Center for Cancer/Cancer Hospital, Chinese Academy of Medical Sciences and Peking Union Medical College, Beijing, 100021, China.

^3^Department of Obstetrics and Gynecology, Department of Pharmacology, The Robert H. Lurie Comprehensive Cancer Center, Northwestern University Feinberg School of Medicine, Chicago, IL, 60611, USA.

**Corresponding Authors**

H.C. ([chenhongyan@cicams.ac.cn](mailto:chenhongyan@cicams.ac.cn))

Z.L. ([liuzh@cicams.ac.cn](mailto:liuzh@cicams.ac.cn)).

**This PDF file includes:**

Materials and Methods

Supplementary Text

Figures. S1 to S8

**Materials and methods**

**Antibodies and reagents**

The antibodies used for immunoblotting (IB), immunoprecipitation (IP) and immunohistochemistry (IHC) were as follows: β-actin, 1:4000 (IB; Sigma-Aldrich, A5316); USP11, 1:5000 (IB; Bethyl Laboratories, Inc, A301-613A), 1:50 (IHC; Sigma HPA003103); BIP, 1:1000 (IB; Cell Signaling Technology, #3177); BIP (IP; Proteintech, 11587-1-AP); 1:200 (IHC; Cell Signaling Technology, #3177); Flag-tag, 1:1000 (IB; Cell Signaling Technology, #8146); Myc-tag, 1:1000 (IB; Cell Signaling Technology, #2276); HA-tag, 1:5000 (IB; Abcam, ab9110). Cycloheximide (CHX; HY-12320) was purchased from MedChemExpress (MCE, NJ, USA), MG132 (C2211) and Chloroquine (CQ) were purchased from Sigma-Aldrich (St. Louis, MO, USA).

**Immunohistochemistry and survival analysis**

Paraffin-embedded in situ tumor tissue blocks were collected from 70 patients with ovarian cancer. This study was approved by the ethical committee of the hospital, and informed consent was obtained from each enrolled patient. Tissue microarrays were stained with anti-USP11 and BIP antibodies. The images were captured by Aperio ScanScope (Leica, Nussloch, Germany). For survival analysis, patients were stratiﬁed according to USP11/BIP expression, Overall Survival (OS) was calculated from the date of surgery to death or the end of follow-up. Patients with recurrent ovarian cancer were categorized as either platinum-sensitive or platinum-resistant based on a platinum-free interval of less than or more than 6 months. IHC staining was quantified by *H*-score = Σ (pi × $\mathcal{i}$), pi stands for the percentage of positively stained cells (0-100%), and $\mathcal{i}$ stands for the staining intensity (0: negative; 1: weak; 2: medium; 3: strong). Kaplan–Meier survival curves were generated and analyzed using the log-rank test.

**Cell culture and siRNA transfection**

HEK293T cells were derived from American Type Culture Collection (ATCC) and maintained in Dulbecco’s modified Eagle’s medium (DMEM) supplemented with 10% fetal bovine serum (FBS). The ovarian cancer ES2 cells were purchased from ATCC and maintained in RPMI 1640 supplemented with 10% FBS. The ovarian cancer 3AO cells were purchased from the Cell Bank of the Chinese Academy of Sciences (Shanghai, China) and maintained in RPMI1640 supplemented with 10% FBS. ON-TARGETplus SMARTpool siRNAs for USP11 and Non-targeting were purchased from Dharmacon. Transient transfection of siRNAs was performed following the standard protocol of Lipofectamine 2000 Reagent. SiRNA sequences targeting USP11 are as follows: GCGCACAGCUGCAUGUCAU, GAGAAGCACUGGUAUAAGC, GGACCGUGAUGAUAUCUUC and GAAGAAGCGUUACUAUGAC.

**Plasmid construction**

The cDNAs of USP11 and BIP were cloned into the pLVX-IRES-Neo vector (#632181, Clontech, CA, USA). The enzymatically inactive USP11^C318A^ mutant vector and deletion mutants of USP11 were preserved in our lab. shRNA oligoes were cloned into the pSIH1-puro vector (#26597, Addgene). The shRNA sequences targeting USP11 were as follows: sh2, 5′-GAGAAGCACUGGUAUAAGC-3′; and sh4, 5′-GAAGAAGCGUUACUAUGAC-3′. The shRNA sequences targeting BIP were as follows: sh1, 5′-GAGCGCATTGATACTAGAAAT-3′; sh2, 5′-AGATTCAGCAACTGGTTAAAG-3′. All constructs were verified by DNA sequencing.

**Cell proliferation assay**

A total of 3×10^3^ cells were suspended in culture medium and seeded in 96-well plates. The medium was replaced with drug-supplemented medium 12h later. After another 12h of drug treatment, Cell Counting Kit-8 (CCK8) reagents were added at a dilution of 1:10 and co-incubated for 1h. Absorbance values were then measured at a wavelength of 450 nm using a microplate reader (BioTek, Winooski, VT, USA).

**Flow cytometry analysis**

3×10^5^ cells were seeded in six-well plates and incubated at 37°C for 24h. Then, the medium was replaced with fresh medium containing CBP for another 24h. According to the recommended protocol from the apoptosis detection kit (LIANKE, China), the apoptosis rates were analyzed by the Annexin-V-FITC and PI double-staining method through flow cytometry (BD Biosciences, San Jose, CA, USA).

**Quantitative real-time PCR**

Total RNA was extracted from cells using TRIzol reagent (Thermo Scientific, Grand Island, NY, USA) and then reverse transcribed to cDNA by a Quantscript RT Kit (Tiangen, Beijing, China). The qRT-PCR analysis was performed on a StepOnePlus Real-Time PCR system (Applied Biosystems (ABI), Foster City, CA, USA). Relative gene expression levels were quantified using the 2-ΔΔCt method and the results were normalized to those of the housekeeping gene GAPDH. The GAPDH primers are as follows: forward, 5′-CCGGGAAACTGTGGCGTGATGG-3′; reverse, 5′-AGGTGGAGGAGTGGGTGTCGCTGTT-3′; The USP11 primers are as follows: forward, 5′-TATAAGCAGTGGGAGGCATACG-3′; reverse, 5′-ATGACCTTGCGTTCAATGGGT-3′.

**Co-Immunoprecipitation (Co-IP) and Western Blotting**

Cells were lysed with RIPA buffer supplemented with a protease inhibitor cocktail (Roche, Basel, Switzerland) for 30 min on ice and then were centrifuged at 13000 rpm for 30 min. The supernatants were collected and the concentrations were measured by a BCA Kit (Thermo Scientific). For Co-IP assays, equal amounts of total protein were incubated with anti-FLAG M2 affinity gels (A2220, Sigma) or anti-BIP antibody (11587-1-AP, Proteintech) overnight at 4°C. Then the beads were washed by cell lysis buffer for three times. The samples were separated on 8%-10% gels depending on the molecular weights of the proteins, and then the proteins were transferred onto polyvinylidene difluoride membranes (Merck Millipore, Billerica, MA, USA). Blots were blocked and incubated overnight at 4°C with the primary antibodies. Secondary antibodies were incubated and then washes were done. Blots were developed with chemiluminescent reagents from Pierce.

**Sliver staining and mass spectrometry**

Cellular extracts from ES2 cells stably overexpressing Flag-USP11 and control cells were incubated with anti-Flag M2 affinity gel (A2220, Sigma) and eluted with 1× loading buffer. The proteins were collected and resolved on 10% sodium dodecyl sulfate-polyacrylamide gel electrophoresis. Gel was silver-stained by PierceTM Silver Stain for Mass Spectrometry (24600, Thermo Scientific) according to the manufacturer’s protocol. The differential protein bands were excised from gel for mass spectrometry analysis.

***In vivo* deubiquitination and ubiquitination assays**

HA-Ub and Flag-BIP were co-transfected with empty, Myc-USP11 or Myc-USP11^C318A^ into HEK293T cells. The cells were treated for 6h with 20mM MG132 before they were harvested. Proteins were immunoprecipitated to isolate ubiquitinated BIP by an anti-HA antibody. Endogenous BIP was immunoprecipitated by anti-BIP antibody and subsequently immunoblotted by anti-ubiquitin antibody in USP11-depleted 3AO and control cells.

**Animal experiments**

All animal protocols were approved by the Animal Care and Use Committee of the Chinese Academy of Medical Sciences Cancer Hospital. For subcutaneous xenografting, 5×10^6^ ES2 cells were subcutaneously implanted into 6-week-old female BALB/c nude mice from Vital River (Beijing, China). Four days after implantation, the mice were randomly divided into two groups and were treated with saline or CBP (50mg/kg). The tumor lengths and widths were measured by a caliper. The tumor volume was calculated with the formula 0.5 × length × width^2^. After tumors had grown for the designated time, all the mice were euthanized, and the tumors were harvested and weighed.

**Statistics**

Statistical significance was calculated using an unpaired Student’s *t*-test. Correlation analysis was performed in R (version 3.6.3). Functional assays *in vitro* were performed at least three times. Differences which p $\leq$ 0.05 was considered statistically significant. All the data analysis was performed using GraphPad Prism version 8.2.0 (San Diego, CA, USA).

**Supplementary Figures and Figure Legends**


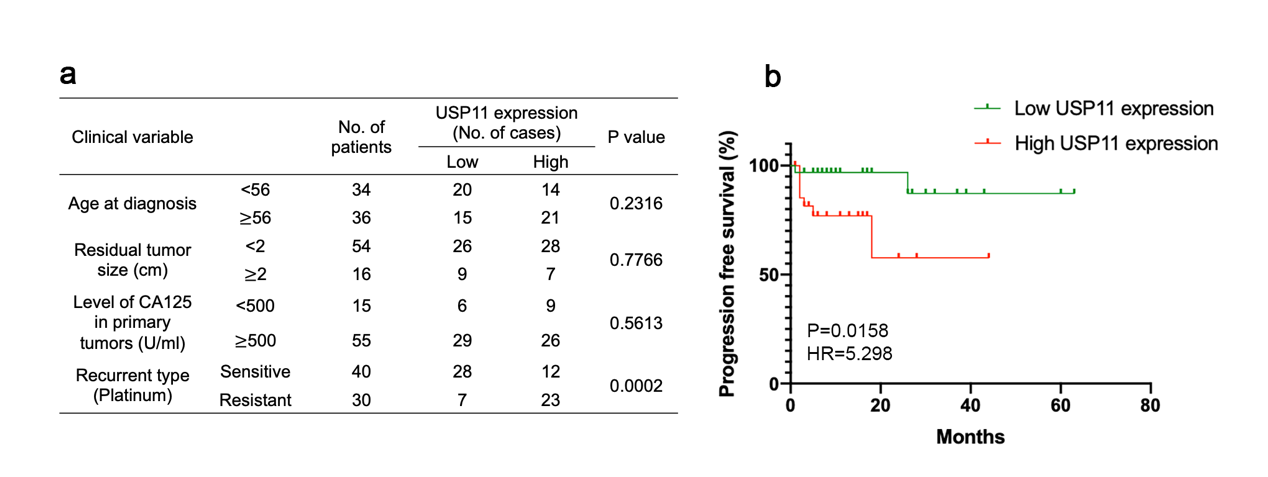


**Fig. S1 High USP11 expression is correlated with poor prognosis and chemoresistance in ovarian cancer. a** The USP11 expression was determined using IHC in 40 chemosensitive and 30 chemoresistant tumor tissue specimens. The expression of USP11 was evaluated by H-score. **b** Kaplan-Meier analysis of ovarian cancer patients’ progression-free survival grouped by low expression or high expression of USP11. P-values were determined by log-rank test.


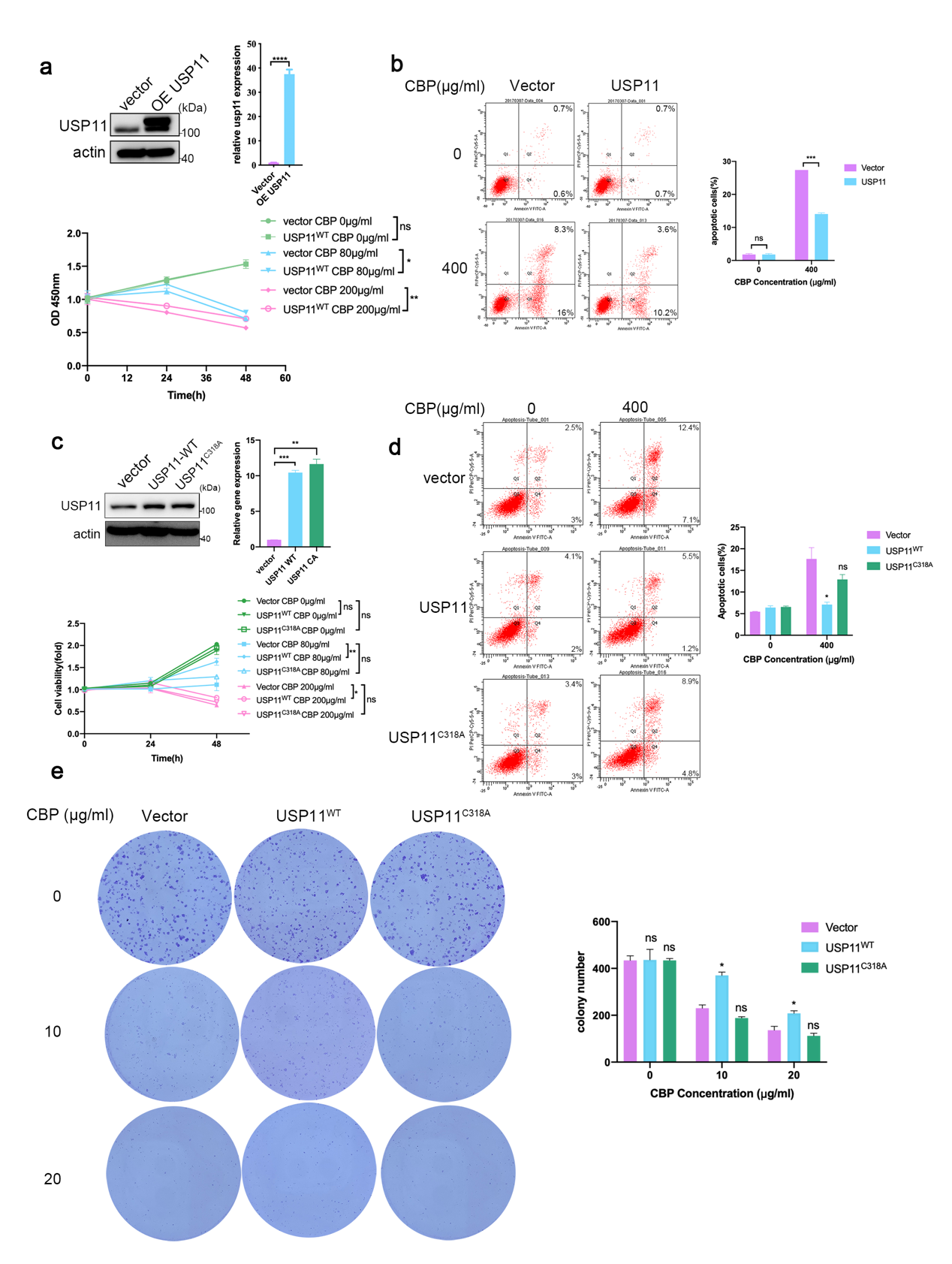


**Fig. S2 USP11 overexpression promotes CBP resistance in ovarian cancer cells.** **a** Overexpression efficiency of USP11 in ES2 cells was determined by Western Blot and qRT-PCR (Upper panel). Cell proliferation detected by CCK-8 assay without or with CBP (80µg/ml or 200µg/ml) treatment (Lower panel). **b** Cell apoptosis detected by Annexin V-PI assay after USP11 overexpression in ES2 cells without or with CBP (400µg/ml) treatment. **c** Overexpression efficiency of USP11^wt^ and USP11^C318A^ in 3AO cells was determined by Western Blot and qRT-PCR (Upper panel). Cell proliferation detected by CCK-8 assay without or with CBP (80µg/ml or 200µg/ml) treatment (Lower panel). **d** Cell apoptosis detected by Annexin V-PI assay in USP11^wt^ or USP11^C318A^ -overexpressed 3AO and control cells without or with CBP (400µg/ml) treatment. **e** Cell survival detected by colony formation assay in USP11^wt^ or USP11^C318A^ -overexpressed 3AO and control cells without or with CBP (10µg/ml or 20µg/ml) treatment. Data in (b, d, e) represent mean ± Standard Deviation (SD) and were analyzed by unpaired two-tailed Student’s *t*-test. Data in (a, b Lower panel) are presented as mean of biological replicates in a representative experiment ± SD and are analyzed by two-way ANOVA with Bonferroni correction. ns=no significant, * *P* < 0.05, ***P* < 0.01, ****P* < 0.001.


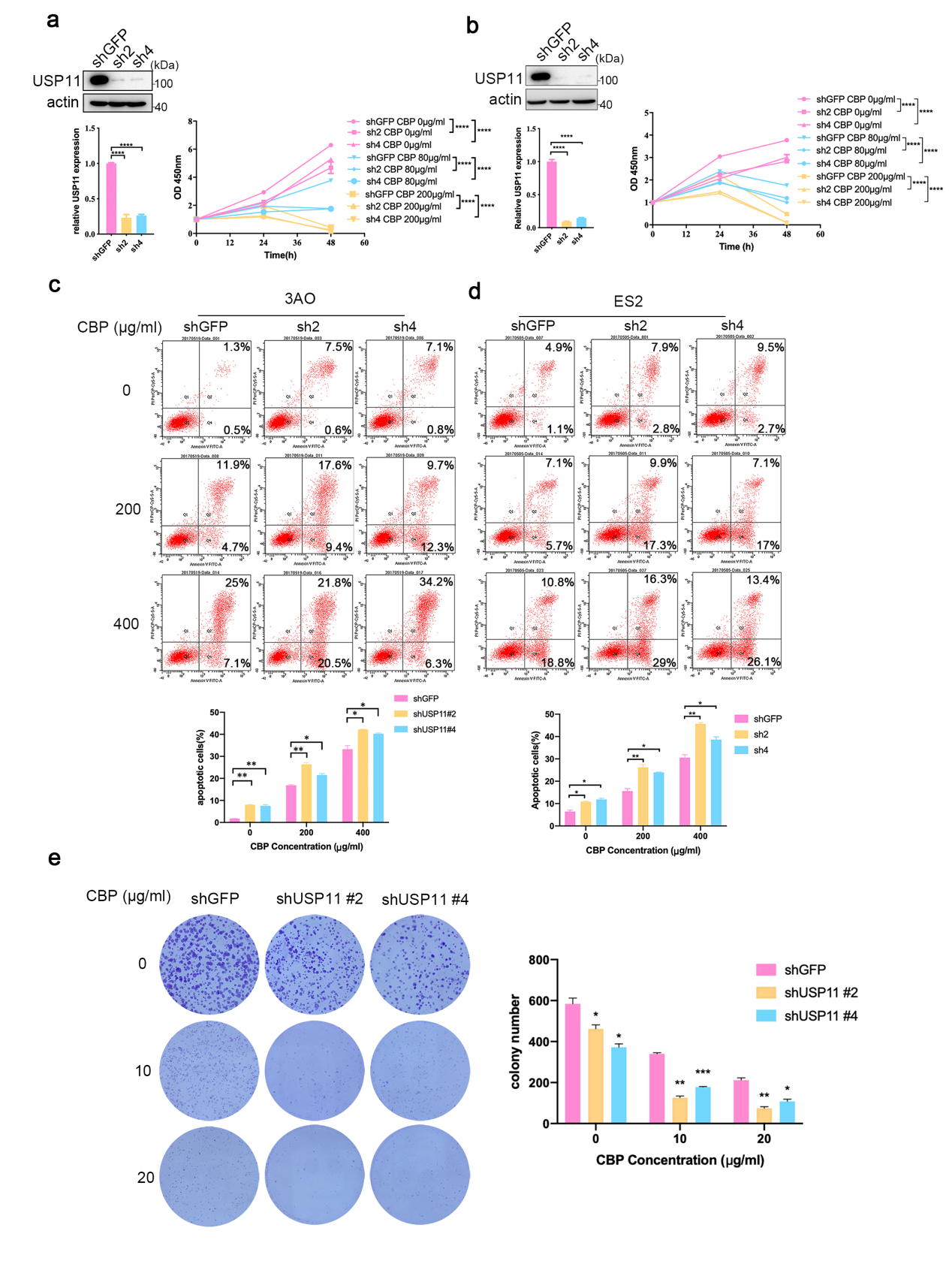


**Fig. S3 USP11 knockdown promotes CBP sensitivity in ovarian cancer cells.** **a&b** Knockdown efficiency of USP11 in 3AO (a) and ES2 (b) was determined by Western Blot and qRT-PCR (Left panel). Cell proliferation detected by CCK-8 assay after USP11 knockdown in 3AO (a) and ES2 (b) cells without or with CBP (80µg/ml or 200µg/ml) treatment (Right panel). **c** & **d** Cell apoptosis detected by Annexin V-PI assay after USP11 knockdown in 3AO (Left panel) and ES2 (Right panel) cells without or with CBP (200µg/ml or 400µg/ml) treatment. **e** Cell survival detected by colony formation assay in USP11-depleted 3AO and control cells without or with CBP (10µg/ml or 20µg/ml) treatment. Representative pictures (Left panel) and quantification (Right panel) were shown. Data in (a, b Left panel, c, d, e) represent mean ± Standard Deviation (SD) and were analyzed by unpaired two-tailed Student’s *t*-test. Data in (a, b Right panel) are presented as mean of biological replicates in a representative experiment ± SD and are analyzed by two-way ANOVA with Bonferroni correction. * *P* < 0.05, ***P* < 0.01, ****P* < 0.001, *****P* < 0.0001.


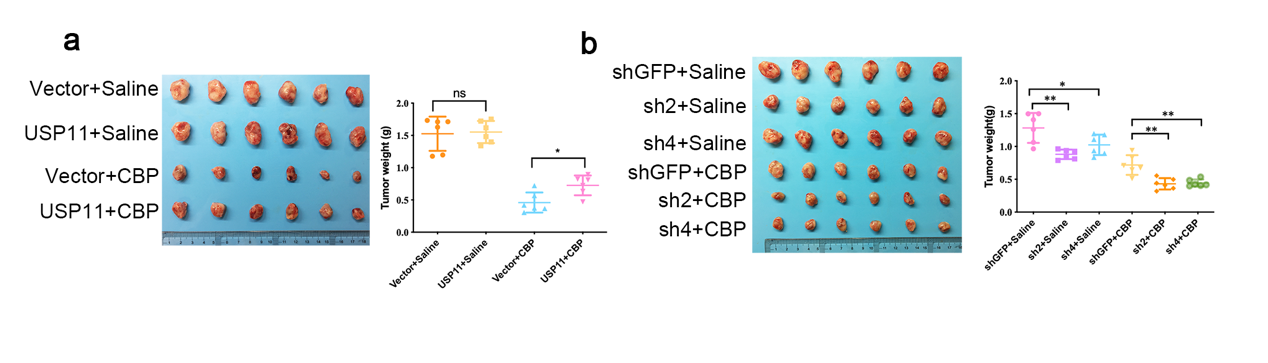


**Fig. S4 USP11 promotes carboplatinum resistance of ovarian cancer cells *in vivo.*** **a** Tumor pictures (Left panel) and tumor weight (Right panel) of USP11-overexpressed ES2 cells and vector control without or with CBP treatment. n = 6 mice per group. **b** Tumor pictures (Left panel) and tumor weight (Right panel) of USP11-silenced ES2 cells and control cells without or with CBP treatment. n = 6 mice per group. Data were analyzed by unpaired two-tailed Student’s t-test. ns=no significant, * *P* < 0.05, ***P* < 0.01.


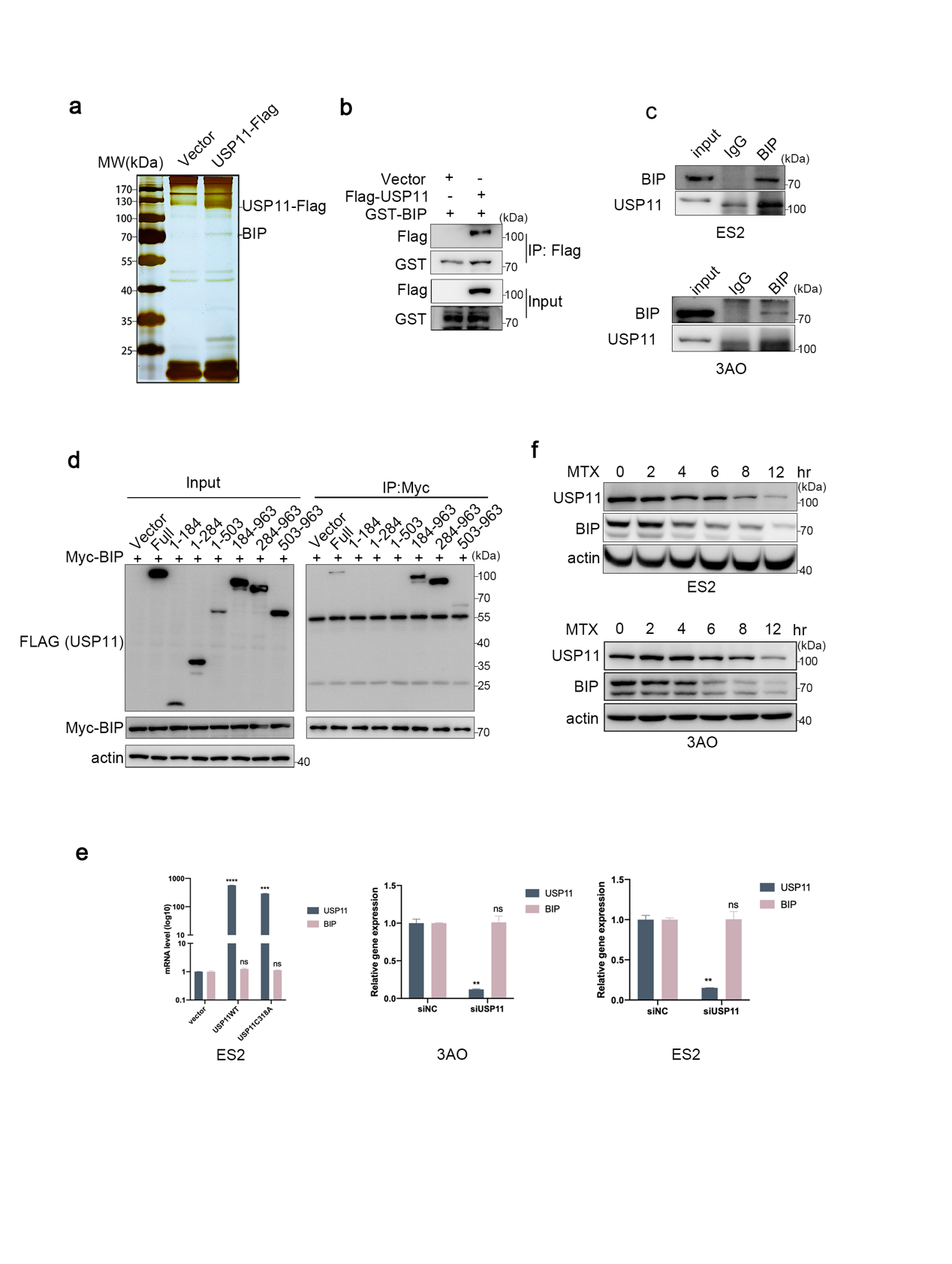


**Fig. S5 USP11 interacts with BIP and regulates BIP protein level.** **a** USP11 stable expression clone was established. Proteins that interacted with USP11 were purified from ES2 cells expressing Flag-tagged USP11 or vector control. Several proteins including BIP were identified in the USP11 protein complex. **b** Interaction between bacterially expressed GST-BIP and Flag-USP11 was demonstrated using GST pull down assay. **c** Endogenous BIP was immunoprecipitated by anti-BIP antibody and subsequently immunoblotted by anti-USP11 antibody in ES2 (upper panel) and 3AO (lower panel) cells. **d** HEK293 cells were co-transfected Myc-BIP with full length USP11 or its deletion mutants into HEK293 cells and Co-IP assays were performed followed by immunoblotting analysis. e The mRNA expression level of USP11 and BIP was detected by RT-qPCR after overexpression of USP11^WT^ or USP11^C318A^ and the knockdown of USP11. f Immunoblotting analysis of BIP expression in ES2 (upper panel) and 3AO (lower panel) cells with MTX treatment at the indicated time points.


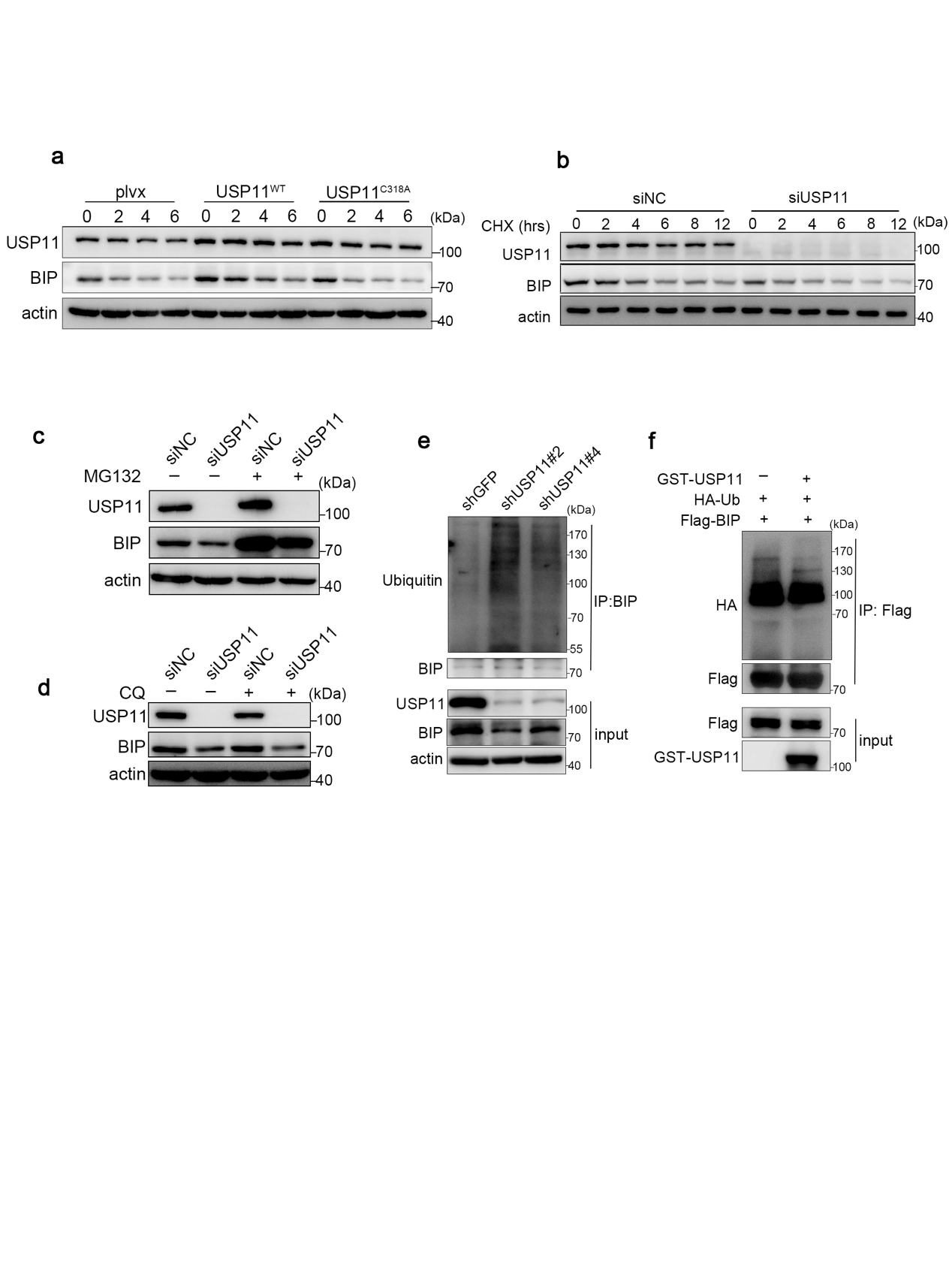


**Fig. S6 USP11 stabilizes BIP via proteasome-mediated pathway. a** Immunoblotting to detect the expression of BIP 3AO cells transfected with Myc-USP11^wt^, Myc-USP11^C318A^ vector control cells with CHX treatment at the indicated time points. **b** Immunoblotting to detect the expression of BIP in ES2 cells transfected with USP11 siRNA and siNC with CHX treatment at the indicated time points. **c&d** 3AO cells transfected with siRNAs targeting USP11 and control were treated with MG132 (c) and CQ (d), Immunoblotting was performed to detect the expression of USP11 and BIP. **e** Endogenous BIP was immunoprecipitated by anti-BIP antibody and subsequently immunoblotted by anti-ubiquitin antibody in 3AO cells with stable USP11 depletion. **f** Bacterially expressed and purified GST-USP11 protein was incubated with cell lysates from HEK293T cells transfected with FLAG-BIP and HA-Ub, and then BIP was immunoprecipitated using anti-FLAG antibody and subsequently immunoblotted by anti-HA antibody.


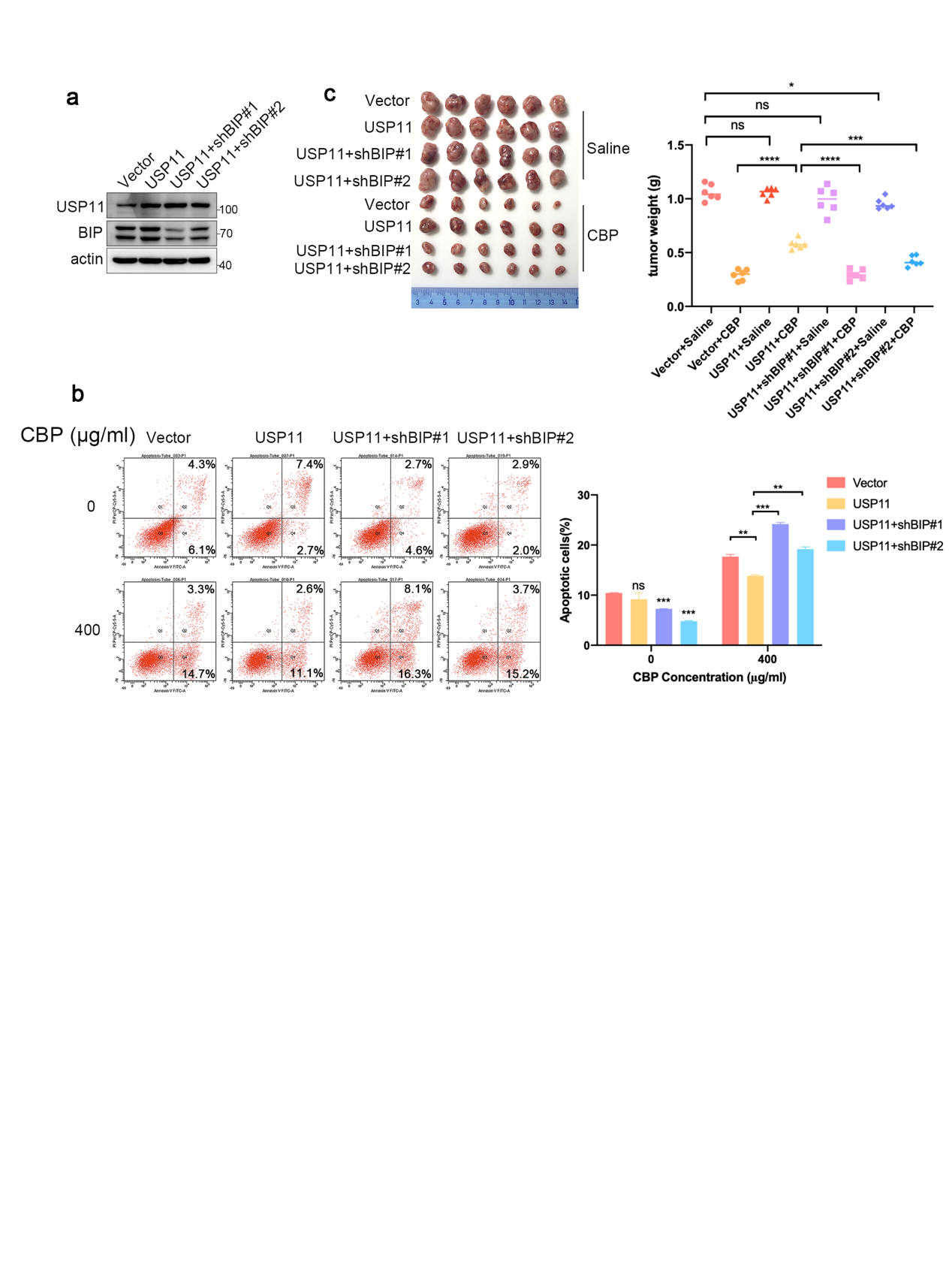


**Fig. S7 USP11 promotes chemoresistance by targeting BIP.** **a** Immunoblotting to detect the expression of USP11 and BIP in ES2 cells transfected with USP11, or USP11 together with shBIP#1 or shBIP#2 and vector control. **b** Cell apoptosis detected by Annexin V-PI assay after USP11 overexpression combined with BIP silencing in ES2 cells without or with (400µg/ml) CBP treatment. **c** Tumor pictures (left panel) and tumor weight (right panel) of ES2 cells expressing USP11*,* USP11 together with shRNAs targeting BIP and vector control without or with CBP treatment. n = 6 mice per group. Data in b&c represent mean ± Standard Deviation (SD) and were analyzed by unpaired two-tailed Student’s t-test. ns=no significant, * *P* < 0.05, ***P* < 0.01. ****P* < 0.001, *****P* < 0.0001.


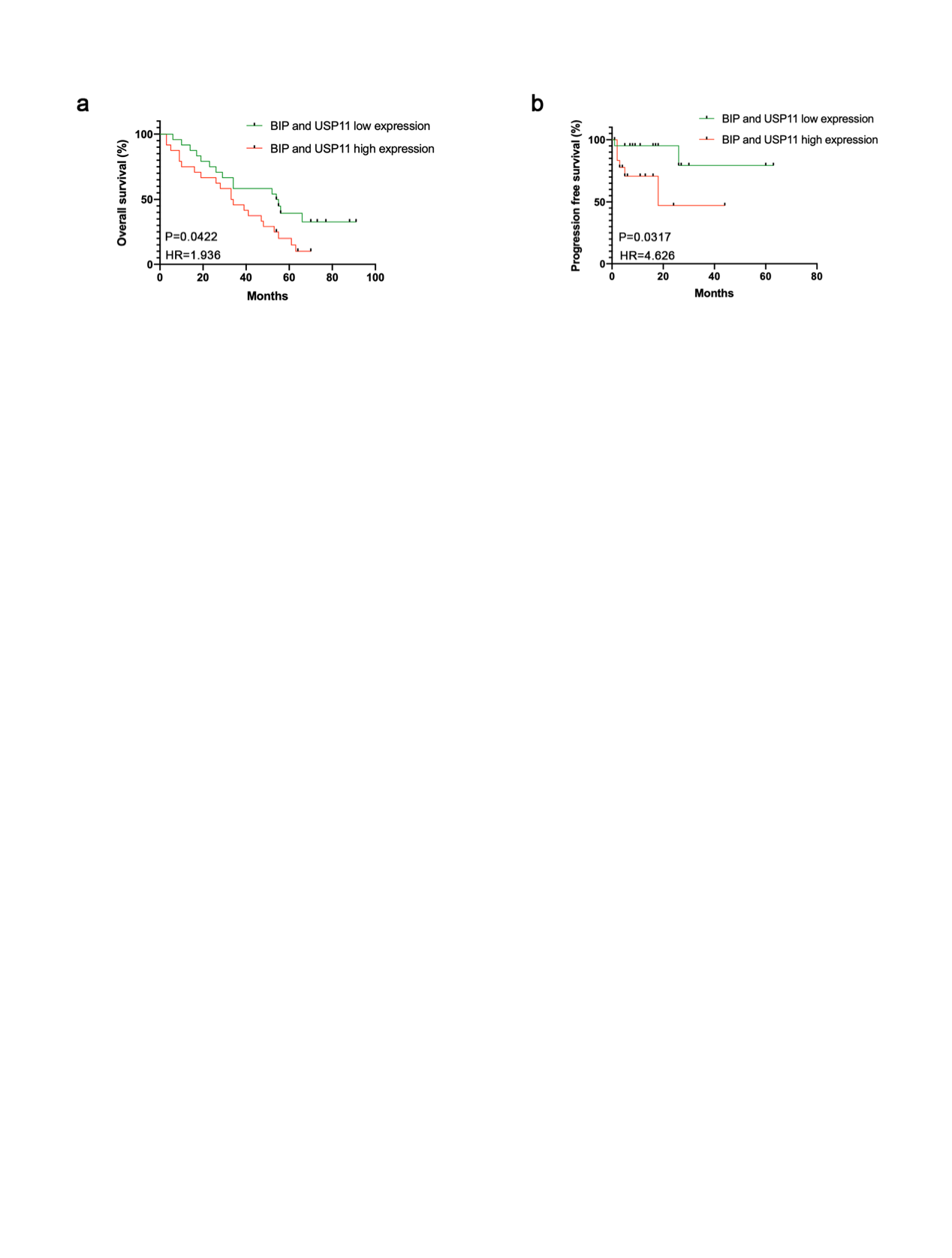


**Fig. S8 The combined expression of USP11/BIP predicts the prognosis of ovarian cancer patients.** Kaplan-Meier analysis of ovarian cancer patients’ overall survival (Left panel) and progression-free survival (Right panel) grouped by low expression or high expression of USP11/BIP. *P*-values were determined by log-rank test.
